# Purple Corn Anthocyanin Affects Lipid Mechanism, Flavor Compound Profiles, and Related Gene Expression of *Longissimus Thoracis et Lumborum* Muscle in Goats

**DOI:** 10.3390/ani11082407

**Published:** 2021-08-14

**Authors:** Xingzhou Tian, Qi Lu, Shengguo Zhao, Jiaxuan Li, Qingyuan Luo, Xu Wang, Yangdong Zhang, Nan Zheng

**Affiliations:** 1 State Key Laboratory of Animal Nutrition, Institute of Animal Science, Chinese Academy of Agricultural Sciences, Beijing 100193, China; tianxingzhou@yeah.net (X.T.); luqi2556728@163.com (Q.L.); zhaoshengguo1984@163.com (S.Z.); 2 College of Animal Science, Guizhou University, Guiyang 550025, China; jiaxuanli8899@163.com (J.L.); natty579@163.com (Q.L.); wx12345678888@163.com (X.W.); 3 Institute of Animal Nutrition and Feed Science, Guizhou University, Guiyang 550025, China; 4 Key Laboratory of Animal Genetics, Breeding and Reproduction in the Plateau Mountainous Region, Ministry of Education, Guizhou University, Guiyang 550025, China

**Keywords:** purple corn anthocyanin, lipid mechanism, flavor compound, gene expression, growing goats

## Abstract

**Simple Summary:**

Natural flavor compounds can stimulate people’s senses of smell and taste; as indicators of food sensory quality, such compounds influence the acceptance by consumers. In addition, natural antioxidants are becoming popular among consumers because they are safe and have no adverse side effects. Purple corn anthocyanins are polyphenolic compounds with natural antioxidant properties that exist widely in nature. Research has shown that anthocyanins can provide extra electrons to the free radicals, preventing lipid oxidation and improving muscle volatile components. However, no information is available concerning the effect of feeding anthocyanin on goat meat volatile compounds. This was the first study to investigate the effects of dietary anthocyanins from purple corn supplementation on lipid mechanism, body composition, volatile compound profiles, and related gene expression in the *longissimus thoracis et lumborum* muscle of goats. The current study indicates that the consumption of purple corn anthocyanin by growing goats improves mutton flavor by decreasing plasma lipid metabolism parameters and by modulating the abundance of several flavor-related genes in the *longissimus thoracis et lumborum* muscle. The results will help to understand the mechanism of action of anthocyanins on the flavor compounds, providing the rationale for anthocyanins regulating mutton flavor through related signaling pathways of ruminants in future studies.

**Abstract:**

The current study aimed to investigate the effect of anthocyanins on muscle flavor compound profiles in goats. Goats in three groups were fed a basic diet or a diet supplemented with 0.5 g/d or 1 g/d anthocyanin-rich purple corn pigment (PCP). Compared to the control group, plasma total cholesterol was significantly decreased (*p* < 0.05) in the anthocyanin groups. The feeding of anthocyanin increased (*p* < 0.05) flavor compound types and total alcohol level, whereas it decreased (*p* < 0.05) total hydrocarbons, aromatics, esters, and miscellaneous compounds in the *longissimus* *thoracis et lumborum* muscle (*LTL*). Adding PCP to the diet enriched (*p* < 0.05) vegetal, herbaceous, grease, and fruity flavors compared to the control group. The 0.5 g/d PCP group had increased (*p* < 0.05) abundance of peroxisome proliferator-activated receptor gamma, but there was a decreased (*p* < 0.05) level of lipoprotein lipase in *LTL*. Collectively, this study indicated that anthocyanin can improve mutton flavor by decreasing plasma lipid parameters and by modulating the abundance of several flavor-related genes of goats.

## 1. Introduction

Flavor is one of the key factors affecting consumers’ choice of food. Currently, a large number of studies have focused on improving flavor compounds of goat meat from the standpoint of animal nutrition. A previous study showed that lipids can easily deteriorate, leading to the reduction of oxidative stability and the production of various volatile components [1]. Once the odor threshold for certain secondary oxidation products is exceeded, consumers may detect aromas of rancidity [2]. Synthetic antioxidants can inhibit lipid oxidation, and these have been widely used in meat production, but they have been discouraged because of their toxic effects. This has led the meat industry to search for new economical and effective natural antioxidants that can replace synthetic antioxidants without adversely affecting the quality of finished products and consumer perceptions [3].

Anthocyanins are natural antioxidants that are widely present in plants. The anthocyanins belong to the class of water-soluble phenolic compounds [4]. Studies have shown that adding anthocyanin-containing feed affected the meat quality in ruminants. For example, Colombo et al. [5] found that the inclusion of anthocyanin-rich purple corn could enhance antioxidant status and help improve unsaturated fatty acids (UFA) in ruminants. The main flavor compounds were alcohols, aldehydes, ketones, esters, benzenes, alkenes and volatile organic acids. The UFA can be easily destroyed by oxygen derived free radicals (FR). Hence, lipids are important flavor compound precursors, especially for the UFA. Specifically, the oxidation of lipid may occur at the double bonds in the UFAs, giving rise to undesirable flavor compounds [6]. It was previously reported that small ruminants receiving purple corn anthocyanins could significantly enhance the antioxidant function [7]. In addition, anthocyanins could regulate gene and protein expression, thus preventing lipid oxidation and improving volatile components [8]. This is because anthocyanins used as FR terminators donate hydrogen to the FRs and thereby stop the oxidation reactions and improve flavor compounds [9]. As mentioned above, anthocyanins had the potential to improve meat volatile components by modulating the lipid mechanism and related gene expression in goats.

To the best of our knowledge, limited information is available regarding the effect of dietary anthocyanin on goat meat volatile compounds. Additionally, we previously confirmed that dietary anthocyanins can be transferred to body tissues, thereby affecting antioxidant functions [10]. We hypothesized that the inclusion of anthocyanin-rich purple corn pigment (PCP) could improve meat volatile compound profiles in goats. The aim of the present study was to investigate the mechanism of anthocyanins affecting volatile compounds in meat through plasma lipid metabolites, body composition, volatile compound profiles, and related gene expression analyses in the *longissimus* *thoracis et lumborum* muscle (*LTL*) of goats.

## 2. Materials and Methods

### 2.1. Experimental Design

This study was designed in agreement with the Inspection Form for Guizhou University, Experimental Animal Ethics (EAE-GZU-2020-7009, Guiyang, China). The feeding trial was conducted in a commercial goat farm (106.198244 E, 28.26403 N, Xishui, China). The experiment lasted 74 days, from 10 July to 24 September in 2020. The preparation period was 14 days, and the formal experimental period was 60 days. A total of 18 Qianbei Ma wether kids (body weight, 21.38 ± 1.61 kg; mean ± standard deviation) were randomly allotted into three groups using a completely randomized design with six replicates per group. The control group was provided with a basal diet, while treatments 1 (LA) and 2 (HA) were supplemented with 0.5 g/d and 1 g/d PCP, respectively. PCP was purchased from Nanjing Herd Source Bio-technology Co., Ltd., Nanjing, China. The anthocyanin composition of PCP was detected according to Tian et al. [11], and PCP had 2619.04 µg/g total anthocyanin concentration in this study. The level of purple corn pigment followed Tian et al. [10]. The PCP was mixed in concentrate, and then mixed with roughage to prepare total mixed rations. All experimental kids were housed in clean individual pens and were provided drinking water *ad libitum*. Equal amounts of rations were provided twice daily at 08:30 and 16:30 for *ad libitum* intake. The nutrient requirements of experimental animals were according to the National Research Council (NRC) [12]. The ingredients and nutrient composition of experimental diets was list in Table 1.

### 2.2. Sample Collection

Approximately 100 g basal diet was collected once weekly and pooled during the experimental time period. The samples were dried at 65 °C in a vacuum oven for 72 h, then ground and passed through a 1-mm sieve.

One day before the end of the experiment, blood samples (approximately 10 mL) were taken at 0 h and 3 h from the jugular vein using a vacuette tube with heparin sodium (Taizhou Qiujing Medical Instrument Co., Ltd., Taizhou, China). The blood samples were transferred to a 1.5-mL tube after centrifugation (Allegra^®^ X-30R Centrifuge, Beckman Coulter, Brea, CA, USA) at 3000× *g* for 15 min at 4 °C and kept at −20 °C until plasma lipid metabolism was analyzed.

Six goats per group were slaughtered at the end of the experiment, and the processing of carcasses was as described by Danforth [13]. Muscle samples were divided into three portions. One aliquot was weighed and put into a freeze dryer (SCIENTZ-18N, Ningbo Scientz Biotechnology Co., Ltd., Ningbo, China) for 72 h to calculate moisture, then ground and passed through a 1-mm sieve until being analyzed for chemical composition. Another aliquot was stored at −20 °C until flavor compounds were detected. The last aliquot was immediately transferred to a 1.5-mL tube with RNA wait (Cat#SR0020, Beijing Solarbio Science and Technology Co., Ltd., Beijing, China) and stored at −80 °C until the gene expression assay was performed.

### 2.3. Chemical Composition

The contents of moisture/dry matter (DM), crude protein (CP), ether extract (EE), ash, calcium (Ca) and phosphorus (P) were measured in feed and muscle as per the method of Association of Official Analytical Chemists (AOAC) [14]. Neutral detergent fiber (NDF) and acid detergent fiber (ADF) were determined according to Van Soest et al. [15]. Gross energy (GE) was analyzed by a calorimeter (WGR-WR3, Changsha BENTE Instrument Co., Ltd., Changsha, China). Each index was run in triplicate.

### 2.4. Plasma Lipid Mechanism

The concentrations of total cholesterol (T-CHO; code No. A111-2-1), triglyceride (TG; code No. A110-2-1), creatinine (Cr; code No. C011-1-1), low-density lipoprotein cholesterol (LDL-C; code No. A113-1-1), and high-density lipoprotein cholesterol (HDL-C; code No. A112-1-1) were determined using commercial kits purchased from Jiancheng Bioengineering Institute (Nanjing, China). All procedures were strictly completed in accordance with reagent instructions.

### 2.5. Flavor Compound Profiles

A total of 4 g of chopped and mixed sample was weighed and transferred to a solid phase microextraction instrument and run in a manual injector with a 2 cm–50/30 μm DVB/CAR/PDMS StableFlex fiber tip. The *LTL* was then subjected to the analysis of volatile compounds using a gas chromatograph (GC; Agilent Technologies, Santa Clara, CA, USA) and tandem mass spectrometer (MS; SCIEX-6500Qtrap; AB Allen-Bradley, Milwaukee, WI, USA) with splitless injecting the sample after headspace extraction-temperature for 60 min at 80 °C. The GC conditions were as follows: the chromatographic column was an Agilent 19091S-436HP-5MSfused silica capillary column (60 m × 250 μm × 0.25 μm). The initial temperature was 40 °C for 2 min, increasing to 180 °C at the rate of 3.5 °C/min, and then to 310 °C at the rate of 10 °C/min. Total run time was 55 min. The temperature of the boil room was 250 °C; the carrier gas was He (99.99%). The pressure before columniation was 15.91 psi; the carrier gas flow rate was 1.0 mL/min, and the solvent delay time was 3 min. The MS conditions were as follows: the ion source was EI ionization; the ionization temperature was 230 °C, the Quadrupole temperature was 150 °C; the energy of ionization was 70 eV, with an emission current of 34.6 μA and multiplier voltage of 1847 v; the interface temperature was 280 °C, and the mass range was 29–500 amu. The peaks in the total ion flow diagram were retrieved by the MS computer data system, and component qualitative analysis was performed via comparison with the standard mass spectra in Nist17 and Wiley275 libraries to determine volatile chemical components. The area normalization method was used to calculate the peak area percentage of each of flavor compound.

### 2.6. Calculation of Relative Odor Activity Value

The contributions of volatile compounds toward the flavor were analyzed by relative odor activity value (ROAV) using the following equation [16]:

ROAV_i_ ≈ C_i_/C_max_×T_max_/T_i_ × 100 (1)

where C_i_ and T_i_ are the relative content and odor threshold concentration, respectively; C_max_ and T_max_ are the highest relative content and odor threshold concentration, respectively. The most important flavor component of ROAV was defined as 100. In general, compounds with ROAV >1 contribute to the aroma, whereas compounds with ROAV < 1 may not.

### 2.7. Gene Expression

Total RNA was extracted from the *LTL* as follows: a 100 mg sample was transferred into a 1.5-mL tube, and 1-mL of RNA extracting solution (Cat. No. G3013; Wuhan Servicebio Technology Co., Ltd., Wuhan, China) was added and run in a homogenizer. The supernatant was immediately transferred to a 1.5-mL tube after centrifugation at 12,000× *g* for 10 min at 4 °C (D3024R; DragonLab, Beijing, China). Next, before centrifugation at 12,000× *g* for 10 min at 4 °C, 250 mL chloroform was added and the tube shaken vigorously, and the mixture was let stand for 3 min. A volume of 400 µL of the supernatant was transferred to a new tube; 320 µL isopropanol (Cat. No. 80109218; Sinopharm Chemical Reagent Co., Ltd., Shanghai, China) was added, and the tube was shaken vigorously and kept at −20 °C for 15 min. The sample was centrifuged at 12,000× *g* for 10 min at 4 °C; the supernatant was removed, and 1.5 mL of 75% ethanol (Cat. No. 10009218; Sinopharm Chemical Reagent Co., Ltd., Shanghai, China) was added to wash the precipitate. The supernatant was removed after centrifugation at 12,000× *g* for 10 min at 4 °C, and the centrifuge tube was placed on a super clean table and aerated for 3 min. The total RNA was dissolved with HyPure^TM^ Molecular Biology Grade Water (Cat. No. SH30538.02; HyClone, Logan, UT, USA) and incubated at 55 °C for 5 min. The concentration and purity of RNA were detected by a NanoDrop 2000 (Thermo Fisher Scientific, Waltham, MA, USA). All sample concentrations were adjusted to 200 ng/μL.

The cDNA synthesis was performed using a Servicebio^®^ RT first strand cDNA synthesis kit (Cat. No. G3330; Wuhan Servicebio Technology Co., Ltd., Wuhan, China) with 20 µL reaction volumes containing 4 μL of 5× reaction buffer, 0.5 μL of oligo (dT)_18_ primer (100 μM), 0.5 μL of random hexamer primer (100 μM), 1 μL of Servicebio^®^ RT enzyme mix, 10 μL of total RNA, and RNase-free water to a final volume of 20 µL.

All primers were designed using the primer 5 software and were synthesized by the Wuhan Servicebio Technology Co., Ltd. (Wuhan, China; Table 2). The three target genes were peroxisome proliferator-activated receptor gamma (*PPARγ*), stearoyl-CoA desaturase (*SCD*), and lipoprotein lipase (*LPL*). The reference gene was glyceraldehyde-3-phosphate dehydrogenase (GAPDH). The real-time PCR amplifications were performed in a 15-µL reaction volume by a StepOnePlus™ real-time PCR system (Applied Biosystems™, Waltham, MA, USA). The reaction system consisted of 7.5 μL of 2 × SYBR Green qPCR master mix (Cat. No. G3320; Wuhan Servicebio Technology Co., Ltd., Wuhan, China), 1.5 μL of forward and reverse primers (2.5 μM), 2.0 μL of cDNA, and 4.0 μL of ddH_2_O. The real-time PCR amplification procedure comprised 10 min at 95 °C for pre-denaturation, 40 cycles of 15 s at 95 °C for denaturation, and 30 s at 60 °C for extension. The fluorescence signal was collected every 0.5 °C from 65 °C to 95 °C. Each sample was performed in triplicate.

### 2.8. Statistical Analysis

All data analysis was performed through the Statistical Analysis System 9.1.3 (SAS Institute, Cary, NC, USA) software using one-way analysis of variance. Six replications were used in the present study, which could make a significance level of 0.05 and a power greater than 0.80. The replicate was considered the experimental unit in all of the statistical analyses. The relative mRNA abundance was measured according to the 2^−ΔΔCt^ method. The average abundance of genes in control data was used as the calibrator. The relationships between gene expression and key volatile compounds of *LTL* were analyzed by Pearson correlation coefficients (r) [17]. *p*-values lower than 0.05 indicated significant differences between samples.

## 3. Results 

### 3.1. Lipid Metabolism

There were no differences (*p* > 0.05) in the plasma TG, LDL-C, or HDL-C among the three groups (Table 3). However, PCP supplementation decreased (*p* < 0.05) the level of plasma T-CHO. In addition, the Cr level in the LA group was significantly lower (*p* < 0.05) than those of the control and HA groups.

### 3.2. Nutrient Composition of Longissimus Thoracis et Lumborum Muscle

No differences (*p* > 0.05) were observed in *LTL* chemical composition for moisture, GE, CP, EE, Ash, Ca, or P among all groups (Table 4).

### 3.3. Flavor Compounds in Longissimus Thoracis et Lumborum Muscle

As shown in Table 5, a total of 41 flavor compounds were detected in this study, including 9 alcohols, 10 aldehydes, 6 ketones, 6 hydrocarbons, 3 aromatics, 4 esters, and 3 miscellaneous compounds. The control, LA, and HA groups detected 27, 35, and 35 flavor compounds, respectively. Compared to the control, the anthocyanin groups showed higher (*p* < 0.05) levels of total alcohols, whereas they displayed lower (*p* < 0.05) levels of total hydrocarbons, aromatics, esters, and miscellaneous compounds. In addition, the HA group had significantly lower (*p* < 0.05) total aldehydes relative to the other groups. The LA group had a significant decrease (*p* < 0.05) in the level of total ketones compared to the controls.

### 3.4. Relative Odor Activity Value

Fourteen key flavor compounds were selected according to the ROAV values (Table 6). Octanal was chosen as the calibration standard because its odor threshold was lowest (0.7). The ROAV values of other compounds were calculated relative to the value for octanal. The key flavor compounds were 1-heptanol (ROAV = 1.711), 1-octen-3-ol (ROAV = 19.742), hexanal (ROAV = 20.025), heptanal (ROAV = 8.061), octanal (ROAV = 100), nonanal (ROAV = 427.875), decanal (ROAV = 1.626), and 2,3-octanedione (ROAV = 2.844) in the control group. Importantly, in addition to the above flavor compounds, the key flavor compounds added 1-hexanol (ROAV = 3.191; ROAV = 3.148) and 1-octanol (ROAV = 1.179; ROAV = 1.565) in the anthocyanin groups.

### 3.5. Gene Expression

**Figure 1:**
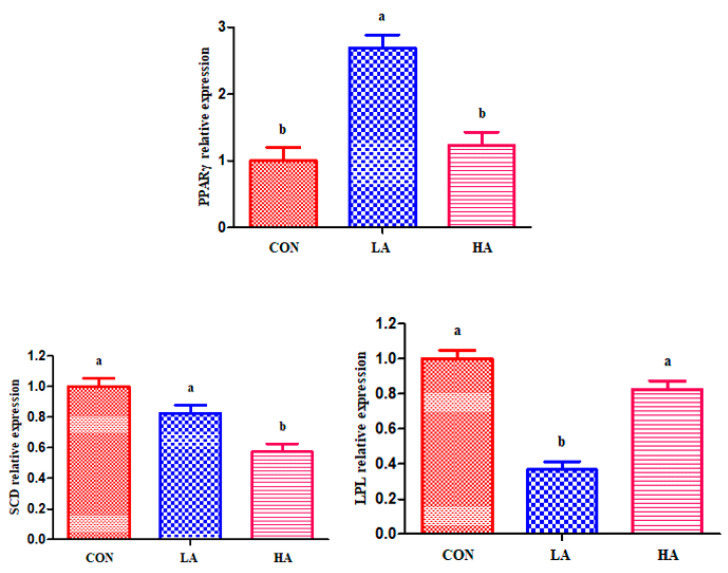
Relative mRNA abundance of genes of *longissimus thoracis et lumborum* muscle in goats. Data reported as least-squares means ± SEM (*n* = 6). Relative quantification of mRNA abundance for each gene was analyzed by the 2^−ΔΔCt^ method, with the control group as the reference expression point. ^a,b^ Different letters indicate significant differences (*p* < 0.05). Abbreviations: LA, basal diet + 0.5 g/d PCP; HA, basal diet + 1 g/d PCP; *PPARγ*, peroxisome proliferator-activated receptor gamma; *SCD*, stearoyl-CoA desaturase; and *LPL*, lipoprotein lipase.

As shown in Figure 1, the abundance of *PPARγ* mRNA was upregulated (*p* < 0.05) in the *LTL* of goats receiving LA relative to the other groups. Goats fed the HA diet showed decreased (*p* < 0.05) *SCD* mRNA abundance compared to those in the control and LA groups. The addition of LA in goats decreased *LPL* (*p* < 0.05) mRNA expression compared with other groups.

### 3.6. Relationship between Gene Expression and Key Volatile Compounds

Significant (*p* < 0.05) negative correlations were observed between *PPARγ* and hexanal and heptanal, while *PPARγ* was positively correlated with octanal (Table 7). There was a significant (*p* < 0.05) positive correlation between the abundance of *SCD* and ethanol. In addition, significant (*p* < 0.05) positive correlations were detected between the mRNA expression of *LPL* and several flavor compounds (ethanol, 1-heptanol, 1-heptanol, (Z)-2-octen-1-ol, hexanal, heptanal, and decanal).

## 4. Discussion

The FRs may initiate the oxidative degradation of lipids, as lipid radicals can react with oxygen and form new FRs such as O_2_^−·^ and ^·^OH, and these can react with TG or free fatty acids (FA) to reinitiate the process in ruminant meat [21]. Anthocyanins may affect the initial processes of lipid hydrolysis, micelle formation, and cholesterol transfer to intestinal cells [22]. Xia et al. [23] reported that adding anthocyanin-rich extract to the diet of mice reduced the concentrations of serum TG and T-CHO, thus improving the lipid profile. In addition, Hosoda et al. [24] demonstrated that the feeding of anthocyanin-rich corn silage tended to lower plasma T-CHO concentration in dairy cows. The postulated mechanism of anthocyanins for inhibition of lipid metabolism was as follows: (1) anthocyanins can inhibit cholesterol synthesis by decreasing the gene expression of 3-hydroxy-3-methylglutaryl coenzyme A; (2) anthocyanins may reduce blood apo B–and apo C-III; and (3) anthocyanins may inhibit cholesteryl ester transfer protein [25]. Yong et al. [26] revealed that purple sweet potato anthocyanins could decrease sterol regulatory element-binding protein 1 level and the expression levels of the target genes acetyl-coenzyme A carboxylase and FA synthase. Similarly, Lee et al. [27] demonstrated that anthocyanins could reduce TG in 3T3-L1 cells. Thus, feeding anthocyanin-rich PCP resulted in a reduction of plasma T-CHO, suggesting that anthocyanins may partly contribute to the actions of lipid metabolites. The autoxidation of lipids or the oxidation of lipids with UFAs leads to the formation of meat flavor compounds. Hence, anthocyanins inhibit plasma lipid metabolism that can modulate the formation of flavor compounds in goat muscle.

Animal meat products are composed of water, protein, fats, and minerals, and nutritional status can directly affect the chemical composition of meat. In the present experiment, no significant differences in nutrient composition were noted among the groups, indicating that anthocyanins had no effect on regulating nutritional components of goats. The reason may be related to the low bioavailability of anthocyanins in animals. In addition, factors affecting the stability of anthocyanin include pH, temperature, oxygen, light intensity, and enzymes [28]. The chemical forms and biological activities of anthocyanins may be different when exposed to different pH and temperatures in the gastrointestinal tract. In addition, the red luteal cation is the most abundant molecular form for anthocyanins when the pH value is less than 2 [29]. Ruminal fluid pH ranged from 7.19 to 7.22 in this study, and this may also negatively affect the absorption of anthocyanins.

Lipid oxidation in meat is one of the main causes of quality loss; oxidation adversely affects flavor and nutritive value, limiting the shelf-life of meat [3]. The lipid hydroperoxides are decomposed into hydroxyl and alkoxy radicals, and then the FA chain adjacent to the alkoxy group splits to produce low molecular weight volatile compounds [9]. Therefore, meat flavor is an important factor affecting the palatability and acceptability of ruminant meat by consumers [30]. Anthocyanins may be transferred from feed to muscle, subsequently affecting the lipid metabolism of muscle [31]. Specifically, anthocyanin is a powerful FR scavenger that can not only effectively prevent the oxidation reactions caused by active FRs but also protect the integrity of lipids [32]. Moreover, the phenolic hydroxyl group of anthocyanins has a strong inhibitory effect on FRs, providing H atoms to FRs and thereby reducing the peroxide value, terminating the reactions of FRs and inhibiting lipid oxidation [33]. As a result, the anthocyanin groups showed high amounts of volatile compounds, perhaps due to the addition of anthocyanins to the goat diet inhibiting lipid oxidation, delaying the decline of meat flavor quality, and enriching the types of flavor substances.

Although lipid oxidation is one of the main reasons for the deterioration of meat products, it is important for the formation of typical aromas in meat products. Alcohols are mainly derived from the lipid oxidation of meat [34]. Of interest, the anthocyanin groups had higher relative contents of alcohols, perhaps because the antioxidant system in goats can meet its own needs. Indeed, lipid peroxidation can easily produce various off-flavor volatile compounds, such as alcohols, ketones, and aldehydes [35]. The polyhydric alcohols in bulk fish oils suppress both oxidation and formation of volatile off-flavors [36]. Hence, the increased alcohol content in meat after feeding anthocyanin may be a manifestation of the enhancement of antioxidant function. However, the mechanism involved is still unclear. Moreover, while an antioxidant may protect against FRs, it could have no effect at all or in certain circumstances may even act as a “pro-oxidant” that generates toxic oxygen species [37]. The flavor compounds play a key role in the meat sensory attributes, but they are also considered important indicators for the stability of oxidative of lambs [38]. It was one of important body protective mechanisms of anthocyanin that regulating the expression of apoptosis-associated protein and antioxidative enzyme by the nuclear factor erythroid 2-related factor 2 (*Nrf2*) signing pathway [39]. Therefore, adding anthocyanin to the diet can improve the antioxidant capacity of muscle and inhibit the excessive oxidation of lipid, reducing the generation of off-flavor and prolonging the shelf life of goat meat. In the present research, the feeding of purple corn anthocyanins showed low total aldehyde and ketone concentrations, suggesting that anthocyanins play a positive role in the aroma of meat.

The volatile compound contents do not reflect their true contributions to the aroma, because these compounds show different odor thresholds, leading to different sensitivity levels for consumers [40]. As such, ROAV was used to express the contribution of volatile compounds to the aroma of goat meat. In addition, the concentration of flavor is determined by the threshold value, and only volatile components with low odor thresholds can make a direct contribution to flavor. Typically, flavor compounds are considered the key volatile components and are considered to significantly contribute to aroma when the value of ROAV is ≥1; when the value is in the range 0.1 ≤ ROAV < 1, the compounds also contribute to the flavor [41]. Of interest, the feeding of anthocyanin increased 1-hexanol and 1-octanol concentrations, as well as they were the key flavor compounds in both anthocyanin groups (ROAV > 1), suggesting that anthocyanins could enrichment of vegetal, herbaceous, grease, and fruity flavors for goat meat. In short, adding PCP to the diet enriched the flavor substances of goat meat, making the flavor of meat more harmonious. Indeed, lipid oxidation has a negative effect on meat, whereas it can help to form pleasant aromas in some cases [42]. Consistent with our results, Prommachart et al. [43] who showed that purple corn anthocyanin extracted residue in cattle diet could decrease lipid oxidation and increase UFA, might improve flavor compounds in meat.

The UFAs react with molecular oxygen to produce unstable compounds via a FR mechanism, and they produce various flavor compounds that include hydrocarbons, aldehydes, ketones, alcohols, esters, and acids, resulting in off-flavors and off-odors in meat [44]. Kortenska and Yanishlieva [45] showed that phenol could inhibit lipid oxidation as a result of the formation of a complex based on the hydrogen bonds between the hydroxy compounds and the phenol antioxidants. The basic chemical structure of anthocyanins is 3,5,7-three hydroxyl-2-phenyl benzopyran, yielding high efficiency in capturing peroxy radicals in the process of lipid oxidation [46]. Hence, the inclusion of anthocyanins can decrease the levels of total hydrocarbons, aromatics, esters, and miscellaneous compounds in goat muscle. Collectively, adding anthocyanins to the goat diet can improve the types and relative contents of meat flavor and can enrich the vegetal, herbaceous, grease, and fruity flavors.

Anthocyanins have been demonstrated to prevent lipid oxidation due to their special properties [11]. Thus, the formation of flavor compounds was verified by detecting gene expression levels of related signaling pathways. The peroxisome proliferator activated receptors (*PPARs*) are nuclear hormone receptors activated by FAs and their derivatives, and they play a central role in lipid homeostasis. Anthocyanins can improve antioxidant capacity by activating the *Nrf2* signaling pathway [47]. The Nrf2 is involved in the *PPARs* pathway because of its mechanisms of action [48]. In addition, anthocyanins may act as *PPARγ* activators, inducing the adipose tissue remodeling and upregulation of peroxisome *PPARγ* gene expression [47]. Therefore, the feeding of 0.5 g/d PCP had significant relative mRNA abundance for *PPARγ*. Of interest, feeding high amounts (1 g/d) of anthocyanins did not increase *PPARγ* gene expression; this may be because anthocyanins have strong antioxidant activity, and the feeding of low amounts of anthocyanins was unable to alleviate oxidative stress and improve antioxidant capacity.

The *SCD* plays an important role in regulating lipogenesis-related gene expression and in modulating mitochondrial UFA oxidation. Nichols et al. [49] demonstrated that citrus flavonoids can reduce plasma lipid levels, enhance glucose tolerance, and inhibit hyperlipidemia and obesity by reducing the mRNA level of the *SCD1* gene in the liver. In addition, cholesterol was the main factor affecting *SCD* activity [50]. Accordingly, the abundance of muscle *SCD* mRNA decreased after feeding goats anthocyanin, perhaps because anthocyanin led to a decrease in plasma T-CHO, inhibiting *SCD* activity. Our observations concur with those of Lee et al. [27], who indicated that anthocyanins suppress lipid accumulation by reducing gene and protein expression levels of lipogenic transcription factors of *PPARγ* and *SCD-1*.

The *LPL* is a key enzyme of lipid metabolism. Its primary function is the hydrolysis of the core TG of circulating chylomicrons and very low-density lipoprotein (VLDL). There are two main sources of FAs involved in the *PPAR* signaling pathway: (1) exogenous sources from circulating FA-albumin complexes or from *LPL*-mediated hydrolysis of plasma VLDL and chylomicrons; and (2) endogenous de novo synthesis [51]. Duivenvoorden et al. [52] showed that mice with increased *LPL* activity had higher fat tissue mass and were more insulin-resistant. Therefore, the feeding of 0.5 g/d PCP had increased abundance of *PPARγ*, and consequently decreased *LPL* gene expression. Consistent with our results, Kowalska et al. [53] demonstrated that anthocyanin-rich cranberries could inhibit lipid metabolism by down-regulation of the mRNA level of *LPL* in the adipose tissue. In short, anthocyanin may change *LTL* flavor compound profiles of goats by activating the expression of *PPARγ* and inhibiting the expression of *SCD* and *LPL.*

Anthocyanins can decrease hepatic lipid accumulation and counteract oxidative stress and hepatic inflammation [54]. The flavor substances derived from the oxidative rancidity of FAs and the addition of anthocyanin in the diet of goats consequently decreased the levels of flavor compounds. In addition, anthocyanin could modulate the expression of related lipid metabolism genes. Interestingly, *SCD* and *LPL* are the key genes that regulate the lipid metabolism in ruminants. Thus, significant positive correlations were detected between *SCD* and ethanol, as well as *LPL* and ehanol, 1-heptanol, 1-heptanol, (Z)-2-octen-1-ol, hexanal, heptanal, and decanal. Anthocyanin-rich plants could alter UFAs biohydrogenation in the rumen, increasing meat content of beneficial FAs, particularly UFA profiles [55]. The UFAs are more prone to lipid oxidation that may lead to more flavor substances in food products. In the current study, significant negative correlations were observed between the mRNA expression of *PPARγ* and hexanal and heptanal, indicating that anthocyanins may modulate the expression of lipid genes and ultimately increase the flavor of meat. Our data provide evidence that lipid mechanisms in plasma and related gene expression levels in *LTL* may be cooperatively involved in anthocyanins regulating the formation of flavor compounds.

## 5. Conclusions

The current study has demonstrated that the inclusion of anthocyanin-rich PCP in growing goats had no effect on the body composition, whereas it could inhibit blood lipid indicators and improve flavor compound profiles in *LTL*. Moreover, anthocyanin-rich PCP could enhance the abundance of *PPARγ* mRNA and could lower the expression levels of the *SCD* and *LPL* genes in the *LTL*. Future research should explore the mechanism of anthocyanins regulating mutton flavor through related signaling pathways.

## Figures and Tables

**Table 1:** Ingredients and nutrient composition of experimental diets (DM basis).

Ingredients (%)	Content	Chemical Composition (%)	Content
Peanut vines	50.00	Dry matter	90.17
White distiller’s grains	10.00	Crude protein	13.82
Soybean residues	10.00	Gross energy (kJ/g)	13.32
Green hay	9.30	Neutral detergent fiber	43.54
Corn	14.00	Acid detergent fiber	29.23
Soybean meal	5.00	Hemicellulose	14.31
Mineral premix ^1^	0.50	Ether extract	2.27
Vitamin premix ^2^	0.50	Organic matter	91.53
NaCl	0.50	Ash	8.47
Limestone	0.20	Calcium	1.04
Total	100.00	Phosphorus	0.18

^1^ Vitamin premix contained (per kg of premix): vitamin A 4,000,000 IU, vitamin D 600,000 IU, vitamin E 25,000 mg, DL-methionine 7000 mg, and L-lysine 5000 mg. ^2^ Mineral premix contained (per kg of premix): Cu 1300 mg, Fe 1000 mg, Zn 1575 mg, and Mn 595 mg.

**Table 2:** Primer sequences used for real-time PCR amplifications in this study.

Gene ^1^	Primer Sequences (5′ to 3′)	Accession Number	Product Size (nt)
*PPARγ*	(F) CATTTCCGCTCCGCACTAC	NM_001285658.1	234
	(R) TGGAACCCTGACGCTTTATCC		
*SCD*	(F) GTTCCAGAATGACGTTTTTGAATGG	NM_001285619.1	175
	(R) CGGATAAATCTAGCGTAGCACCC		
*LPL*	(F) ATTTAACTATCCCCTGGGCAATG	XM_013966067.2	212
	(R) ACCCCCTGGTGAATGTGTGT		
*GAPDH*	(F) GCCCTCTCAAGGGCATTCTA	XM_005680968.3	81
	(R) AGGTAGAAGAGTGAGTGTCGC		

^1^*PPARγ*, peroxisome proliferator-activated receptor gamma; *SCD*, stearoyl-CoA desaturase; *LPL*, lipoprotein lipase; *GAPDH*, glyceraldehyde-3-phosphate dehydrogenase; F, forward; and R, reverse.

**Table 3:** Effect of purple corn anthocyanin on plasma lipid in goats ^1^.

Item ^2^		Group		SEM	*p*-Value
	Control	LA	HA		
T-CHO (mmol/L)	5.00 ^a^	3.85 ^b^	3.34 ^b^	0.2487	0.0006
TG (mmol/L)	0.52	0.54	0.53	0.0284	0.9101
Cr (μmol/L)	74.91 ^a^	60.53 ^b^	73.89 ^a^	4.0951	0.0624
LDL-C (mmol/L)	1.57	1.52	1.52	0.0723	0.8302
HDL-C (mmol/L)	3.30	3.60	3.54	0.1100	0.1656

^1^^a,b^ Different letters within a row indicate significant differences (*p* < 0.05). Values represent the means of six replicates (*n* = 6). ^2^ LA, basal diet + 0.5 g/d PCP; HA, basal diet + 1 g/d PCP; SEM, standard error of mean; T-CHO, total cholesterol; TG, triglyceride; Cr, creatinine; LDL-C, low-density lipoprotein cholesterol; and HDL-C, high-density lipoprotein cholesterol.

**Table 4:** Effect of purple corn anthocyanin on nutrient composition of *longissimus* *thoracis et lumborum* muscle in goats ^1^.

Item (%) ^2^		Group		SEM	*p*-Value
	Control	LA	HA		
Moisture	73.05	74.32	73.06	0.7561	0.4427
GE (kJ/g)	21.72	22.68	22.88	1.0207	0.7083
CP	21.22	20.57	21.54	0.4413	0.3079
EE	11.05	11.14	11.01	0.9605	0.9956
Ash	6.19	6.79	6.03	0.3177	0.2895
Ca	0.40	0.34	0.34	0.0195	0.0809
P	0.26	0.22	0.23	0.0354	0.7060

^1^ Values represent the means of six replicates (*n* = 6). ^2^ LA, basal diet + 0.5 g/d PCP; HA, basal diet + 1 g/d PCP; SEM, standard error of mean; GE, gross energy; CP, crude protein; EE, ether extract; Ca, calcium; and P, phosphorus.

**Table 5:** Effect of purple corn anthocyanin on flavor compounds of *longissimus* *thoracis et lumborum* muscle in goats ^1^.

Item (%) ^2^		Group		SEM	*p*-Value
	Control	LA	HA		
**Alcohols (9)**					
Ethanol	2.04 ^b^	8.55 ^a^	3.12 ^b^	0.3212	<0.0001
1-butanol	ND	0.07 ^b^	0.19 ^a^	0.0120	0.0022
1-pentanol	1.62	1.54	1.86	0.1193	0.2198
1-hexanol	0.17 ^b^	2.08 ^a^	2.06 ^a^	0.1013	<0.0001
1-heptanol	0.75 ^c^	1.64 ^b^	2.11 ^a^	0.1262	0.0008
1-octen-3-ol	1.82 ^c^	3.86 ^b^	5.43 ^a^	0.1539	<0.0001
2-ethyl-1-hexanol	2.11 ^a^	1.16 ^b^	2.13 ^a^	0.1738	0.0119
(Z)-2-octen-1-ol	0.17 ^b^	0.65 ^a^	0.80 ^a^	0.0750	0.0025
1-octanol	10.10 ^b^	13.58 ^a^	10.26 ^b^	0.6600	0.0161
Subtotal	15.05 ^b^	27.11^a^	27.29 ^a^	1.1629	0.0004
**Aldehydes (10)**					
3-methyl-butanal	ND	0.09	0.10	0.0070	0.2293
Acetaldehyde	0.98 ^a^	ND	0.08 ^b^	0.1105	0.0045
Pentanal	ND	0.29	ND	-	-
Hexanal	8.29 ^c^	17.71 ^a^	15.54 ^b^	0.3865	<0.0001
Heptanal	2.22 ^b^	4.07 ^a^	3.91 ^a^	0.1720	0.0005
(Z)-2-heptenal	ND	0.16	0.19	0.0256	0.3811
Octanal	6.46 ^a^	5.07 ^b^	5.10 ^b^	0.2106	0.0052
(E)-2-octenal	ND	0.23 ^a^	0.11 ^b^	0.0167	0.0065
Nonanal	39.43 ^a^	30.67 ^b^	30.34 ^b^	0.3985	<0.0001
Decanal	0.30 ^b^	0.45 ^a^	0.49 ^a^	0.0389	0.0304
Subtotal	57.68 ^a^	58.75 ^a^	55.87 ^b^	0.5207	0.0214
**Ketones (6)**					
Acetone	0.66 ^a^	ND	0.31 ^b^	0.0816	0.0397
2,3-butanedione	ND	ND	0.13	-	-
2-pentanone	ND	0.07	0.13	0.0177	0.0806
2-heptanone	ND	ND	0.37	-	-
2,3-octanedione	3.14 ^a^	1.39 ^b^	2.18 ^b^	0.2424	0.0066
6-methyl-5-hepten-2-one	ND	0.30	0.48	0.0491	0.0534
Subtotal	3.79 ^a^	1.76 ^b^	3.61 ^a^	0.3577	0.0127
**Hydrocarbons (6)**					
1,1-diethoxy-ethane	ND	0.03	ND	-	-
Octane	0.50	ND	ND	-	-
Nonane	ND	0.11	0.12	0.0130	0.9049
1-nitro-hexane	1.60 ^a^	0.55 ^b^	1.20 ^a^	0.1430	0.0058
Dodecane	0.26 ^a^	0.14 ^b^	0.25 ^a^	0.0153	0.0026
Tetradecane	1.22 ^a^	0.34 ^b^	0.43 ^b^	0.0684	0.0002
Subtotal	3.58 ^a^	1.29 ^b^	1.89 ^b^	0.1787	0.0003
**Aromatics (3)**					
Ethylbenzene	0.38 ^a^	0.09 ^b^	0.17 ^b^	0.0280	0.0009
Styrene	0.62 ^a^	0.15 ^b^	ND	0.0390	0.0010
o-xylene	0.55 ^a^	0.28 ^b^	0.22 ^b^	0.0390	0.0022
Subtotal	1.54 ^a^	0.52 ^b^	0.39 ^b^	0.0383	<0.0001
**Esters (4)**					
Ethyl acetate	ND	0.10	ND	-	-
Hexanoic acid, ethyl ester	ND	0.30 ^a^	0.05 ^b^	0.0221	0.0015
Carbamodithioic acid, diethyl-, methyl ester	4.16 ^a^	0.77 ^b^	0.67 ^b^	0.0817	<0.0001
9-octadecenoic acid (Z)-, methyl ester	1.06	ND	ND	-	-
Subtotal	5.22 ^a^	1.17 ^b^	0.73 ^b^	0.1388	<0.0001
**Miscellaneous compounds (3)**					
Carbon disulfide	6.06 ^a^	1.02 ^b^	0.79 ^b^	0.1384	<0.0001
Oxime-, methoxy-phenyl-_	ND	0.30 ^b^	0.52 ^a^	0.0231	0.0025
2-pentyl-furan	0.49	0.45	0.21	0.1520	0.4222
Subtotal	6.55 ^a^	1.76 ^b^	1.52 ^b^	0.1884	<0.0001

^1^^a–c^ Different letters within a row indicate significant differences (*p* < 0.05). Values represent the means of six replicates (*n* = 6). ^2^ LA, basal diet + 0.5 g/d PCP; HA, basal diet + 1 g/d PCP; SEM, standard error of mean; and ND; not detected.

**Table 6:** Relative odor activity values of critical flavor compounds of *longissimus* *thoracis et lumborum* muscle in goats ^1^.

**Name of Compound ^2^**	Odor Threshold (μg/kg)	Odor Description	Literature	ROAV		
				Control	LA	HA
Ethanol	520	NF	19	0.043	0.227	0.083
1-hexanol	9	Vegetal, herbaceous	18	0.205	3.191	3.148
1-heptanol	4.8	Fat flavor	19	1.711	4.708	6.022
1-octen-3-ol	1	Mushroom, earthy	16	19.742	53.105	75.217
(Z)-2-octen-1-ol	13	Grease, fruity	16	0.046	0.224	0.274
1-octanol	120	Grease, fruity	16	0.916	1.179	1.565
Hexanal	4.5	Grassy and fishy	16	20.025	54.318	47.744
Heptanal	3	Fishy	16	8.061	18.698	17.945
Octanal	0.7	Fat, lemon, green	18	100	100	100
Nonanal	1	Grease and grassy	16	427.875	424.049	419.828
Decanal	2	Citrus	16	1.626	3.097	3.359
2,3-octanedione	12	Creamy	20	2.844	1.599	2.553
Carbon disulfide	210	NF	19	0.314	0.067	0.052
2-pentyl-furan	6	Fruity aroma	20	0.894	0.998	0.481

^1^ Odor description refers to the literature [16,18,19,20]. Only flavor components with ROAV ≥ 0.1 in one of the three group samples are presented. ^2^ LA; basal diet + 0.5 g/d PCP; HA; basal diet + 1 g/d PCP; and NF; not found.

**Table 7:** Pearson correlation coefficients between key volatile compounds and gene expression of *longissimus* *thoracis et lumborum* muscle in goats ^1^.

Item ^2^	*PPARγ*	*SCD*	*LPL*
Ethanol	−0.5689	0.7052 *	0.7599 *
1-hexanol	0.3272	−0.6146	−0.5435
1-heptanol	−0.6140	−0.3168	0.7830 *
1-octen-3-ol	−0.5506	−0.4336	0.7323 *
(Z)-2-octen-1-ol	−0.4814	−0.1500	0.8746 **
1-octanol	−0.3095	−0.5766	0.3358
Hexanal	−0.7299 *	0.1437	0.9354 ***
Heptanal	−0.7307 *	−0.0057	0.8861 **
Octanal	0.7443 *	0.0137	−0.8250 **
Nonanal	0.5984	0.0892	−0.9174 ***
Decanal	−0.4527	−0.2811	0.6936 *
2,3-octanedione	0.5060	−0.2555	−0.8111 **
Carbon disulfide	0.6195	0.0782	−0.9262 ***
2-pentyl-furan	0.0778	0.4142	−0.1854

^1^ Only flavor components with ROAV ≥ 0.1 in one of the three group samples are presented. * *p* < 0.05, ** *p* < 0.01, *** *p* < 0.001. ^2^ *PPARγ*, peroxisome proliferator-activated receptor gamma; *SCD*, stearoyl-CoA desaturase; and *LPL*, lipoprotein lipase.

## Data Availability

Data is contained within the article.
